# Illness scripts in nursing: Directed content analysis

**DOI:** 10.1111/jan.15011

**Published:** 2021-08-11

**Authors:** Jettie Vreugdenhil, Donna Döpp, Eugène J. F. M. Custers, Marcel E. Reinders, Jos Dobber, Rashmi A. Kusukar

**Affiliations:** ^1^ Amsterdam UMC, LEARN! Research Institute for Learning and Education Faculty of Psychology and Education Vrije Universiteit Amsterdam the Netherlands; ^2^ Amstel Academie Amsterdam UMC Amsterdam the Netherlands; ^3^ Centre for Research and Development of Education University Medical Centre Utrecht Utrecht the Netherlands; ^4^ Department of Family Medicine Amsterdam UMC Amsterdam the Netherlands; ^5^ Amsterdam School of Nursing Center of Expertise Urban Vitality Faculty of Health Amsterdam University of Applied Sciences Amsterdam the Netherlands; ^6^ Amsterdam UMC Faculty of Medicine Faculty of Psychology and Education LEARN! Research Institute for learning and education Vrije Universiteit Amsterdam the Netherlands

**Keywords:** advanced practice, clinical judgement, clinical reasoning, directed content analysis, education, illness scripts, nurses, nursing

## Abstract

**Aims:**

To explore the possible extension of the illness script theory used in medicine to the nursing context.

**Design:**

A qualitative interview study.

**Methods:**

The study was conducted between September 2019 and March 2020. Expert nurses were asked to think aloud about 20 patient problems in nursing. A directed content analysis approach including quantitative data processing was used to analyse the transcribed data.

**Results:**

Through the analysis of 3912 statements, scripts were identified and a nursing script model is proposed; the medical illness script, including *enabling conditions*, *fault* and *consequences*, is extended with *management*, *boundary*, *impact*, *occurrence* and *explicative statements*. Nurses often used *explicative statements* when pathophysiological causes are absent or unknown. To explore the applicability of Illness script theory we analysed scripts’ richness and maturity with descriptive statistics. Expert nurses, like medical experts, had rich knowledge of *consequences*, *explicative statements* and *management* of familiar patient problems.

**Conclusion:**

The knowledge of expert nurses about patient problems can be described in scripts; the components of medical illness scripts are also relevant in nursing. We propose to extend the original illness script concept with *management*, *explicative statements*, *boundary*, *impact and occurrence*, to enlarge the applicability of illness scripts in the nursing domain.

**Impact:**

Illness scripts guide clinical reasoning in patient care. Insights into illness scripts of nursing experts is a necessary first step to develop goals or guidelines for student nurses’ development of clinical reasoning. It might lay the groundwork for future educational strategies.

## 1 INTRODUCTION

The optimal strategies for fostering the development of nursing students’ clinical reasoning in the academy or in clinical placements still seem unclear (Brown Tyo & McCurry, 2019; Greenwood, 2000). Nurses are increasingly called on to make rapid judgements under conditions of uncertainty due to the rise in acute hospitalized, chronically ill and older patients. In addition, lengths of stay have decreased, while patients suffer from more complex problems, with the accompanying risk of serious deterioration (Johansen & O’Brien, 2016; Lasater, 2011; Levett‐Jones et al., 2010; Purling & King, 2012). Holder (2018) states that flawed reasoning leads to flawed care. For these reasons, considerable attention is paid in clinical placements to preparing nursing students for clinical reasoning. Reasoning in real‐life practice may be influenced by issues related to the individual student, the reasoning task and clinical teaching.

In spite of all the research on clinical reasoning, important questions about educational strategies and the development of clinical reasoning in nursing students during clinical placements still lack evidence‐based answers (Cappelletti et al., 2014; Hunter & Arthur, 2016). In medical education research, illness script theory provides a possible framework for the development of reasoning skills. This theory is based on information processing and memory organization; people tend to organize repeated experiences and connect perceptions if they seem related or happen simultaneously in schemes or scripts (Custers, 2015; Holder, 2018). Whether and how illness script theory is applicable to nursing is unknown. In this study, we explore the potential of illness script theory for nursing, as it might consequently offer a potential scientific basis for designing teaching methods for clinical reasoning.

## 2 BACKGROUND

Nurses’ clinical reasoning can be described as ‘a complex process that uses cognition, metacognition and discipline‐specific knowledge to gather and analyse patient information, evaluate its significance and weigh alternative actions’ (Simmons, 2010, p. 1151). Clinical reasoning, in nursing, as in other health professions, is context‐dependent and domain‐specific and reflects scientific and clinical knowledge (Durning et al., 2011; Hayes Fleming, 1991; Liberati et al., 2016; Malterud, 2001; Simmons, 2010).

Illness script theory proposes that experts’ reasoning is guided by knowledge structures in the memory (scripts), which explains why medical experts are able to quickly interpret complex situations and predict how they might evolve (Charlin et al., 2007; Custers, 2015; Lubarsky et al., 2015). The theory states that illness scripts develop through experience with real patients (Charlin et al., 2007), which explains changes in memory performance, information processing, decision‐making and the decreasing use of biomedical knowledge in growing expertise (Custers, 2015; Custers et al., 1998). Illness scripts play a role in recognizing, comparing, contrasting and predicting the course of a disease (Lubarsky et al., 2015). This theory has been applied in medical and advanced nursing education, in classroom and clinical settings (Banning, 2008; Kassirer, 2010; Lee et al., 2010; Lubarsky et al., 2015).

**FIGURE 1:**
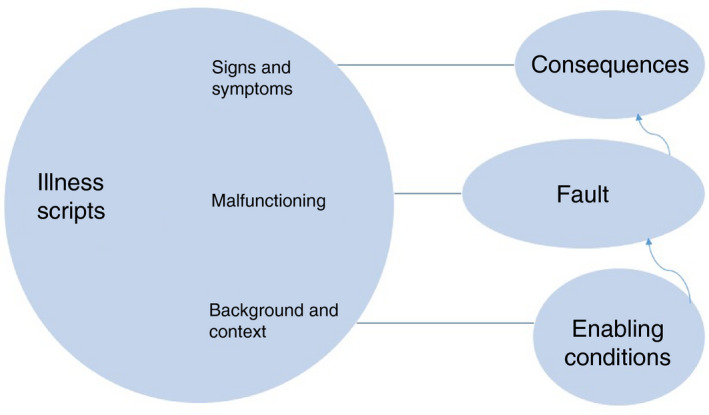
Original structure of illness script (based on Feltovich & Barrows (1984))

Illness scripts have been studied as a possible explanation for professional development and as a concept (Yazdani & Hoseini Abardeh, 2019). As a concept, an illness script is a specific script based on patient encounters, representing clinical knowledge in three components. The original illness script components are as follows:

- *Enabling conditions*: patient features like age or occupation and epidemiological factors that influence the probability of a disease;
- *Fault*: the causal pathophysiological process and disturbed body functions;
- *Consequences*: signs and symptoms; the results of a fault (Figure 1) (Custers, 2015; Feltovich & Barrows, 1984; van Schaik et al., 2005).

The illness script components have been expanded over the years by researchers to include, for example management, environment and a miscellaneous category to improve the fit with actual clinical practice (Custers et al., 1998; Keemink et al., 2018; van Schaik et al., 2005). Strasser and Gruber (2015) investigated script formation of mental health counsellors. In this field, *fault* knowledge is most often not causal or related to body functions. Hence, Strasser and Gruber split the *fault* component into *theoretical concepts* (theory‐based statements) and *explicative statements*, statements that define and explain a problem.

These previous studies have raised questions about how nurses’ clinical knowledge is structured and stored. Therefore, our research question was: *How well does illness script theory describe nurses’ experience*‐*based knowledge?* Clarity about the concept of illness scripts in expert nurses is a necessary first step to develop goals or guidelines for student nurses’ development of clinical reasoning. It might lay the groundwork for future educational strategies.

## 3 THE STUDY

### 3.1 Aims

This study aims to explore the possible extension of the illness script theory used in medicine to the nursing context. We hypothesize that the knowledge of nurses with experience and know‐how, grounded in retrieved patient encounters, can be described by script‐like structures and that the components of nurses’ scripts are analogous to the medical illness scripts, including *enabling conditions*, *consequences*, *patient management* and *fault*.

### 3.2 Design

The chosen methods are based on the studies of Custers et al., (1998) and Strasser and Gruber (2015), who investigated illness scripts of physicians and counsellors. Likewise, we conducted a qualitative, interview study to provide for think‐aloud protocols, which are analysed with deductive directed content analysis (Hsieh & Shannon, 2005; Kao & Parsi, 2004). The purpose of the method of directed content analysis is to validate or extend a theory or framework (Hsieh & Shannon, 2005). This method stems from a naturalistic paradigm and allows for coding interview data from think‐aloud protocols and transforming qualitative data into descriptive quantitative data to find supporting or non‐supporting evidence for illness script theory in nursing (Fetters et al., 2013; Hsieh & Shannon, 2005). The think‐aloud method is selected as a proven effective approach to verbalize cognitive processes like knowledge; it provides rich data related to participants’ clinical reasoning (Offredy & Meerabeau, 2005; Simmons et al., 2003). We decided to investigate our research question among expert nurses because of their greater understanding of clinical situations and patient responses (Offredy & Meerabeau, 2005; Simmons et al., 2003).

### 3.3 Sample/participants

The setting of this research was a large academic hospital (Amsterdam UMC) in The Netherlands. We recruited expert critical care and oncology/haematology nurses from adult intensive care units and oncology/haematology wards with postgraduate specialty qualifications. Based on the unit managers’ selections and recommendations (years of experience in the unit), we approached 18 purposefully sampled nurses with more than 10 years’ experience as a specialized nurse by email to invite them to participate in our study. With their education and experience, we consider them as experts.

### 3.4 Data collection

#### 3.4.1 Materials

**FIGURE 2:**
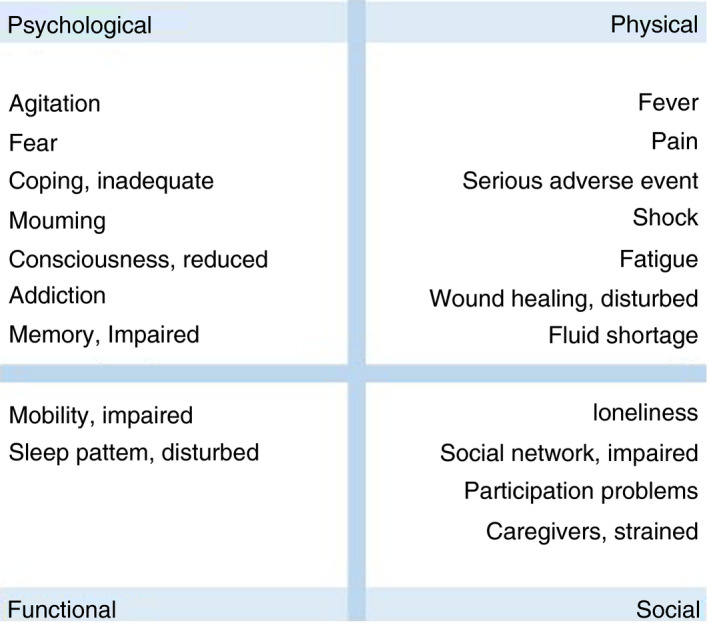
Selected patient problems [Colour figure can be viewed at wileyonlinelibrary.com]

To acquire a list of general, relevant and prevalent patient problems (PPs) in nursing, we selected 20 problems from the Dutch nursing patient problem list, which were identified by nurses in 2012 (Schuurmans et al. (2012). The 20 PPs represent the four areas of human functioning: physical, psychological, social and functional. We added two multidisciplinary problems (shock and serious adverse events) because of the hospital setting (Figure 2) (Hodgetts et al., 2002; Paans & Müller‐Staub, 2015; Schuurmans et al., 2012).

#### 3.4.2 Procedures

The PPs were presented in a PowerPoint presentation in random order to the participants to avoid the order effect. We piloted the presentation and the main question in one interview, and no changes were made thereafter. The interviewer (main researcher) is a specialized neurology nurse, nurse educator and epidemiologist trained in interview techniques, who is known to some participants but held no professional relationships with them at the time. The presentation opened with a worked‐out think‐aloud example about nausea to explain the requested task. The interviewer asked the participants to sequentially work on the 20 different PPs in individual interview sessions of 30 min maximum. The participants were encouraged to tell all they knew about each problem and patients with these problems. The main question was ‘What can you tell me about a patient with …’ The interviewer encouraged the nurses to elaborate on what they usually observe, do, expect and think with probing questions like ‘What do you see?’ or ‘How do you notice it?’ We were interested in the type of information nurses have stored in memory, not in accuracy or comprehensiveness. The interviews, which took place between September 2019 and March 2020, were audio‐recorded and transcribed verbatim.

### 3.5 Ethical considerations

The study was deemed exempt from approval by the Medical Ethical Review Board of an academic hospital. The nurses were invited to participate on a voluntary basis, and informed about the study background by the interviewer, and they signed an informed consent letter. The participants were advised that they could withdraw from the study at any time. All transcripts were anonymized before analysis. Permission to store and process the study data was obtained from the hospital.

### 3.6 Data analysis

Directed content analysis is a highly structured, theory‐based process of deductive coding and analysing (interview) data. The think‐aloud protocols were segmented into statements, these are units with relevant (nursing) information (Custers et al., 1998). Using ATLAS.ti, the first author coded the statements into a category system adapted from Custers et al., (1998) (Figure 1). The intended categories or codes derived from this reference were *fault*, *enabling conditions* and *consequences*. We extended this original category system with the code *management* (Keemink et al., 2018; Monajemi et al., 2012), with the assumption that also for nurses management knowledge is part of their expertise. Like Strasser and Gruber (2015) and with the same assumption that not all PPs can be related to causal, bio‐physical knowledge, we added *explicative statement* to the category system. *Explicative statements* and *fault* together explain or express an understanding of a PP’s origin. Statements that could not be categorized according to this model were clustered and open coded. The frequencies of statements per illness script component were calculated in Excel, along with the number of statements per problem and script component.

### 3.7 Rigour

The first author has had prolonged engagement with hospital nurses and the language they use, which ensures the study's credibility.

The transcribed statements were read and re‐read before coding in several rounds. To ensure confirmability, two randomly selected transcribed interviews were coded independently (DD and JV) (Faucher et al., 2012; Hoffman et al., 2009; Keemink et al., 2018). Differences in coding decisions of the two researchers were discussed to define the codes and adjust the coding decisions. We defined the study sample as expert nurses (with a specialized qualification and >10 years of experience) to enhance transferability. Our data's dependability is reinforced by the 20 different PPs, and confirmability is assured by comparing the codes to earlier studies and the literature. The data were finalized through consensus after discussion in the full research team. All audio recordings, transcripts and coding decisions were recorded in a coding log and ATLAS.ti (Korstjens & Moser, 2018; Nowell et al., 2017).

## 4 FINDINGS

### 4.1 Qualitative

Seven nurses from oncology/haematology units and six nurses from intensive and medium care units participated; all were trained as specialized nurses and had over 10 years’ work experience in their units. Due to the COVID‐19 crisis, the last two planned interviews were cancelled, one nurse withdrew due to illness and two did not reply. All nurses talked extensively about the 20 PPs during the interviews. The think‐aloud protocols of 13 interviews were transcribed and segmented into statements and coded into the above‐mentioned category system.

Not all statements could be coded in these five categories. We decided to include in the *management* category statements about plans for additional tests, interviews and observations. Additionally, we clustered the remaining quotes into three extra categories: *impact*, *boundary* and *occurrence*. Table 1 lists the results of this coding procedure with characteristic quotes and references to previous studies.

**TABLE 1:** Codes, descriptions, references, and quotes of participants (B‐N)

Category	Origin	Reference	Description	Quote
Fault	Original model	(Custers, 2015; Custers et al., 1998; Feltovich & Barrows, 1984)	Statements about the causes of the problem, the pathophysiological processes, anatomy or behaviour	‘hypernatremia’ (L) ‘allergic reaction to any medicament’ (N)
Consequences	Original model	(Custers, 2015; Custers et al., 1998; Feltovich & Barrows, 1984)	Statements about key features of a problem, signs and symptoms, test results and scores	‘Responds very slowly’ (K) ‘That your metabolic is completely disrupted’(N)
Enabling conditions	Original model	(Custers, 2015; Custers et al., 1998; van Schaik et al., 2005)	Statements about patient background information like age, gender, the context and epidemiological aspects like exposure or risk factors that influence the probability of a problem	‘I recently cared for a young woman’(E) ‘Often in ENT patients’(F)
Explicative statement	Extended model	(Strasser & Gruber, 2015)	Statements about factors that explain or can be associated with the problem. The problem ‘can be traced back to’	‘When the pain is not under control, people are less mobile’ (J) ‘And it is a fact that if you start giving chemo the wound, the healing is bad’ (I)
Management	Extended model	(Keemink et al., 2018; Monajemi et al., 2012)	Statements about treatment or intervention plans and decisions, expressions of planning additional tests, interviews or observations	‘Always encourage getting out of bed’(M) ‘You will check the short‐term memory’(B)
Boundaries	Open coding	(Baxter & Brumfitt, 2008; Liberati et al., 2016)	Statements about the boundaries of the domain of nursing actions and expertise	‘And I see my role not so much to solve it, but to pass it on to the people who have the expertise’(C) ‘you make that plan together with the doctors’(D)
Impact	Open coding	Impact (Blondon et al., 2017; Tanner, 2006) Engagement (Berg et al., 2006)	Statements about how the patient or the nurse are affected by the symptoms or the problem	‘therefore a much longer rehabilitation time if someone is in pain, he is obstructed in carrying out all activities’ (B) ‘So that was very difficult to deal with as a team’(G)
Occurrence	Open coding	(van Schaik et al., 2005)	Statements about how common a problem is	‘we see a lot of sad people’ (J) ‘in theory they have no wounds here’ (H)

The three new components were based on the participants’ verbalized experiences. The nurses described the influences of problems on patients’ lives and how problems affected themselves as caregivers (*impac*t). They discussed their daily practice in multidisciplinary teams and the necessity to consult other team members (*boundary*) to provide optimal patient care. The nurses also explained the relation between context and PPs, which we coded as *occurrence*.

### 4.2 Quantitative

The think‐aloud protocols resulted in 3912 statements. The coded statements were summarized in ATLAS.ti in a code co‐occurrence table to measure frequencies and proportions. The mean number of PPs discussed was 17, with a mean of 289 statements per interview. The frequencies and proportions of the components are listed in Table 2, as well as the PPs with the highest and lowest proportions in each component.

**TABLE 2:** Frequencies and proportions of nursing script categories

Script components	Frequency	Proportion % (range)	Low‐high
Consequences	1241	32 (12.4–42.4)	Mobility (impaired)–addiction
Management	808	21 (10.9–27.8)	Serious adverse events–mobility (impaired)
Explicative statements	660	17 (6.6–27.9)	Fluid shortage–sleep pattern (disturbed)
Enabling conditions	417	11 (1.5–18.3)	Addiction–mobility (impaired)
Fault	331	8 (0.5–18.1)	Caregivers (strained)–wound healing (disturbed)
Impact	207	5 (1.8–11.7)	Fever–social network (impaired)
Occurrence	147	4 (1.9–4.8)	Fatigue–caregivers (strained)
Boundary	88	2 (0.5–6.1)	Memory (impaired)–caregivers (strained)
None	13	0	
Total	3912	100	

At the level of the individual PP, we found differences in the number of statements per PP, which might reflect a script's richness (Keemink et al., 2018). PP Pain elicited the most statements (mean: 26.9 statements), and PP participation elicited the least (mean: 8.1 statements). We also found differences between the 20 PPs in the proportion of statements in the specific script components, which can indicate script maturity (Keemink et al., 2018). For example, the range for the proportion of statements in the script component *enabling conditions* was 1.5% (PP addiction) to 18.3% (PP impaired mobility).

**FIGURE 3:**
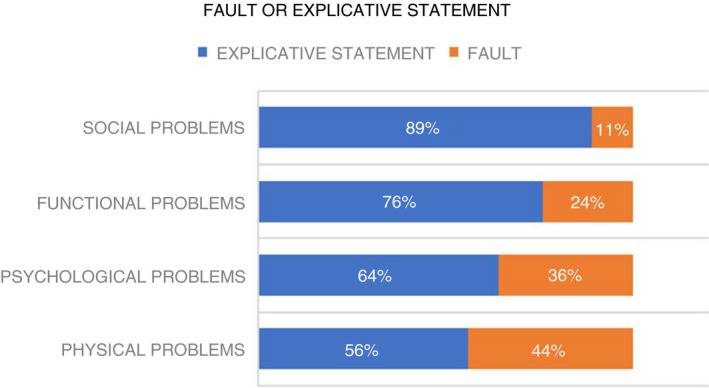
Pattern in explicative statements relative to fault

Since we assumed that nurses would use explicative statements if causal bio‐physical knowledge was irrelevant or not available, we inspected the data to find a pattern in the proportion of statements relating to *fault* or *explicative statements*. In the four areas of human functioning, nurses mentioned relatively more *explicative statements* than *fault* statements when talking aloud about PPs (Figure 3).

## 5 DISCUSSION

**FIGURE 4:**
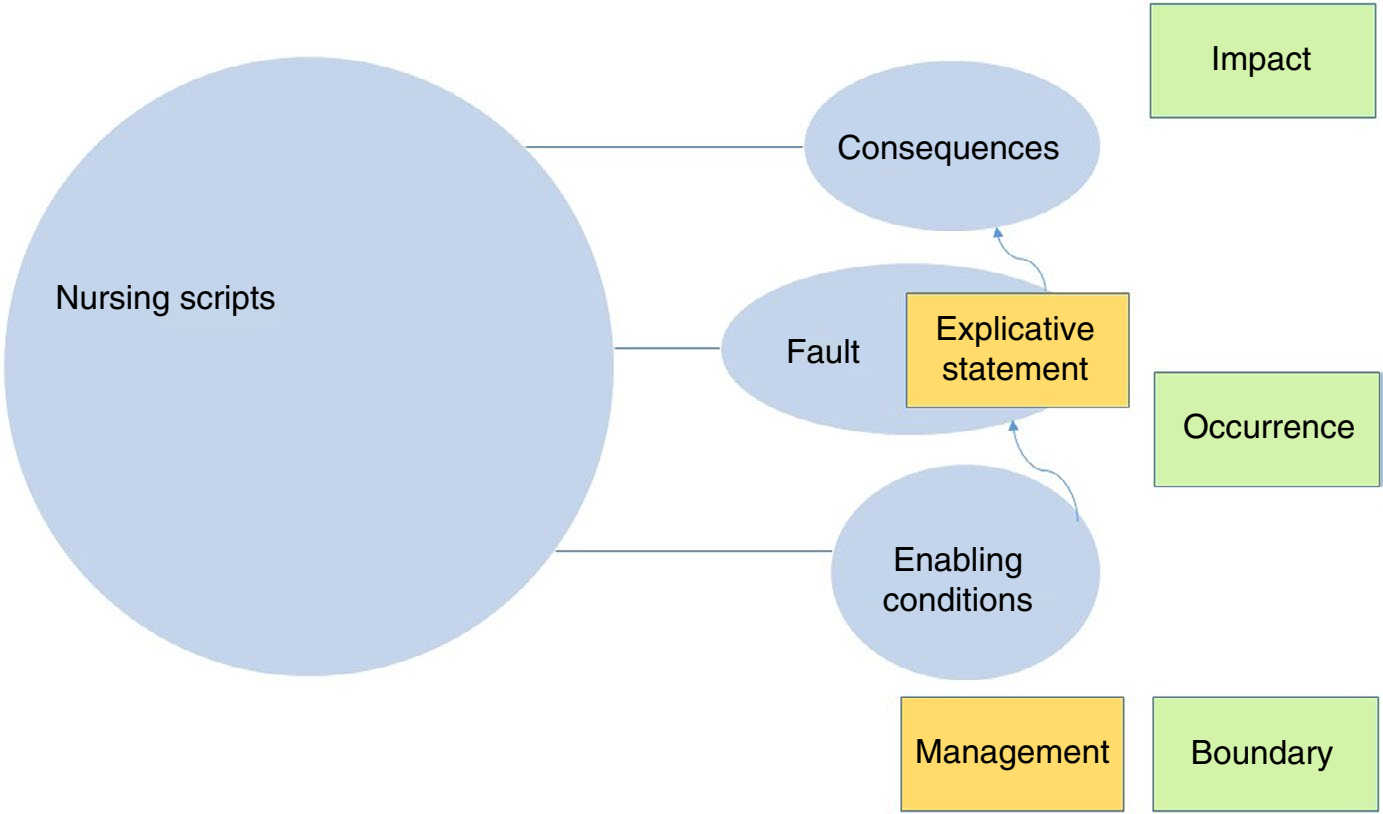
Nursing script model. Blue = original model; yellow = extended model; green = nursing additions

To increase understanding of the nurses’ clinical reasoning, we explored illness script theory applied to nursing. Through directed content analysis, we could identify scripts in the expert nurses’ stories about PPs. In the qualitative results section, we presented the nursing scripts’ building blocks or components. These findings can be depicted in a nursing script model (Figure 4).

In the quantitative analysis, we explored the richness and maturity of the nursing scripts and a pattern in the relationship between *fault* and *explicative statements*.

### 5.1 How can the nursing script model be characterized?

We asked the expert nurses to elaborate on PPs and not on medical diagnoses or illnesses. Nevertheless, we found a script model very similar to previous studies in medical research (Custers et al., 1998; Keemink et al., 2018). The distinct components of nursing patient problem scripts are related to medical illness scripts but have a special nursing flavour.

With regard to the illness script theory's original components, we found the highest frequency of statements about *consequences*, which corresponds with findings in medical studies (Custers et al., 1998; Keemink et al., 2018). A rich palette of signs and symptoms can facilitate recognition (Custers et al., 1998) of a PP and trigger reasoning processes. *Management* statements were mentioned frequently. Monajemi et al., (2012) indicate that (medical) expertise is characterised by scripts with a high proportion of management knowledge. *Enabling conditions* are an important component of illness script theory. In our study, the nurses generally mentioned age, length of hospital stay, certain treatments and clusters of medical diagnoses. Knowledge about *enabling conditions* is acquired through experience. The ability to recognize *enabling conditions* is associated with early and accurate problem identification (Keemink et al., 2018) and is a characteristic of expertise (Schmidt & Rikers, 2007). In our sample, we found an overall proportion of *enabling conditions* of 11%, with variations between the PPs in the proportion of *enabling conditions*. Moreover, as an example, the larger proportion for the PP impaired mobility than for the PP addiction may be explained by the frequency of occurrence of these problems in our setting. This example supports the theory that knowledge about *enabling conditions* is related to growing expertise.

In this study, we found three nursing script model components with small proportions: *boundary*, *occurrence* and *impact*. Van Schaik et al., (2005) suggest incorporating contextual factors like work environment into illness scripts. The components’ *boundary* and *occurrence* in our nursing script model can be considered contextual factors that may also contribute to the context and domain specificity of clinical reasoning. *Impact* is probably the script component that best fits the nursing domain. Nursing concerns the impact of diseases on patients’ lives, health improvement and future functioning (Blondon et al., 2017; Chiffi & Zanotti, 2015). Above that, ‘knowing the patient’ and how a patient responds to a condition is a prerequisite for reasoning (Tanner, 2006). Significant in nursing clinical judgement is also ‘what the nurse brings to a situation’, which includes perceptions, values and opinions, which we also coded as *impact* (Tanner, 2006).

The nursing knowledge of a PP’s origin is captured in this study in both *fault* and *explicative statements*, which is possibly the most interesting result of this study. Pathophysiological malfunctioning is the content of the original *fault* component, and explanations or associations with behaviour or circumstances were coded in this study as *explicative statements*. According to illness script theory and evidence, experts rely less on *fault* knowledge and more on *consequences* and *enabling conditions* (Keemink et al., 2018; Schmidt & Rikers, 2007). In this study, the frequency of both *fault* and *explicative statements* appears high, which seems to contradict illness script theory. A possible explanation may be found in the descriptive nature of the PP and in the fact that many PPs are associated with several causes or factors. However, more significantly, nurses mentioned explicative statements in all four types of PP, not only in the non‐physical ones. In practice, nurses strive to understand the situation and do not necessarily explain it (Levett‐Jones et al., 2010; Ritter, 2003). Maybe it is this characteristic that is captured by the combination of *fault* and *explicative statements*; both knowledge types are probably necessary to enlarge understanding. Moreover, this might not only concern nursing, as recent medical literature about clinical reasoning argues for the integration of ‘biomedical explaining’ and ‘patient understanding’ (Daly, 2018; Gupta et al., 2019; Malterud et al., 2019).

Thus, combining *fault* and *explicative* statements could make illness script theory more applicable to all health professions and follow contemporary movements in professional attitudes about patient care that ‘call for a shift in clinical care away from underlying disease pathology toward understanding people’ (Gupta et al., 2019, p.49).

### 5.2 Illness scripts in nursing

This study contributes to outlining the features of nursing scripts in nursing clinical reasoning. According to illness script theory, reasoning in patient encounters is guided by individual scripts (Lubarsky et al., 2015). Keemink et al., (2018) state that mature expert scripts have a higher emphasis on *enabling conditions* and *consequences* than on *fault*. We encountered rich descriptions of *consequences* and *explicative statements* but fewer descriptions of enabling conditions by the expert nurses in our sample. We learned from illness script theory that recognizing *consequences* and *enabling conditions* earns a distinct place in clinical teaching to enhance clinical reasoning (Lubarsky et al., 2015). With our description of how our expert nurses think, we might better help our future students (Offredy & Meerabeau, 2005; Simmons et al., 2003). Based on this study, it may be advisable to add knowledge about *explicative statements*, *impact* and contextual knowledge to clinical teaching. In practice, nurse educators and preceptors can help students construct their illness scripts based on everyday patient experiences (Greenwood, 2000). Nursing scripts may offer students a tool to improve their understanding of PPs and thus enhance their clinical reasoning skills on possible explanations and potential deterioration risks (Charlin et al., 2007).

### 5.3 Limitations

This explorative study's methods for data collection and analysis might influence validity and generalizability. We used the think‐aloud protocol for data collection. Although this technique is frequently used to access cognitive processes, the outcomes are influenced by participants’ ability to verbalize and describe their conscious thoughts (Banning, 2008). However, since it is impossible to directly observe cognitive processes, the think‐aloud method is a state‐of‐the‐art method to investigate the content of these processes (Offredy & Meerabeau, 2005). Although the sample size was relatively small, it is in line with other qualitative think‐aloud studies (Hunter & Arthur, 2016; Simmons et al., 2003), and the interviews generated many statements about PPs that represented the four areas of human functioning.

We used directed content analysis as an established method to support or extend an existing theory. A known drawback of this method is that researchers are biased towards finding supportive evidence for the theory (Hsieh & Shannon, 2005). To overcome this bias, we also applied open coding, kept a coding log, and two coders independently double coded 2 out of 13 interviews. This study's combined qualitative and quantitative analysis generated insight into nursing clinical reasoning that enabled us to compose the proposed nursing script model, which needs to be validated in a different nursing sample.

## 6 CONCLUSION

Our aim was to explore the applicability of illness script theory in nursing and extend the scope of illness script theory. Our findings support the two hypotheses: The expertise in PPs of expert nurses can be described in a script, and the components of medical illness scripts—*enabling conditions*, *fault*, *consequences* and *management*—are also relevant in nursing.

We propose to extend the original illness script with the components *explicative statements*, *boundary*, *impact* and *occurrence* to make them specific for nurses. Illness script theory seems applicable to nursing, but in this study, the investigation was limited to the concept of illness scripts. Illness script theory also proposes an explanation of the learning path from novice to expert (Yazdani & Hoseini Abardeh, 2019). Hence, before the impact of this study can be fully exploited, we recommend future research to:

1. Test our findings in a broader sample of nurses and students in and outside hospital to explore the development of scripts from novices to experts;
2. Validate the *explicative statement* component in other health professions;
3. Investigate the stability of nursing scripts: Would the nurses make the same statements again, at another time, with another interviewer?
4. Explore and test clinical teaching strategies based on nursing scripts.

## AUTHOR CONTRIBUTIONS

All authors have agreed on the final version and meet the following criteria.

1. Substantial contributions to conception and design (JV, JD, EC, MR and RK), acquisition of data (JV), or analysis and interpretation of data (JV, DD, JD, EC, MR and RK)
2. Drafting the article and revising it critically (JV, DD, JD, EC, MR and RK)

### PEER REVIEW

The peer review history for this article is available at https://publons.com/publon/10.1111/jan.15011.

## Data Availability

The code co‐occurrence table and raw Excel data can be requested from the first author.
